# Significance of the auditory meatus inferior wall cartilage in the surgical treatment of congenital first branchial cleft anomalies in children

**DOI:** 10.1136/wjps-2023-000645

**Published:** 2023-11-09

**Authors:** Bo Yu, Ruiyang Zhu, Yong Fu, Bin Xu, Lulu Yu, Jing Bi

**Affiliations:** 1 Department of ENT and Head & Neck Surgery, Children's Hospital,Zhejiang University School of Medicine, Hangzhou, China; 2 School of Stomatology, Zhejiang Chinese Medical University, Hangzhou, China

**Keywords:** Otorhinolaryngologic Diseases, Congenital Abnormalities

## Abstract

**Objective:**

To investigate the clinical significance of the inferior wall cartilage of the auditory meatus in surgical treatment of congenital first branchial cleft anomalies (CFBCAs) in children.

**Methods:**

Twenty children diagnosed with CFBCAs who underwent surgery between December 2018 and June 2022 at our hospital were retrospectively analyzed and classified according to their Work lesion type. The guiding significance of the inferior wall cartilage in the surgical treatment of CFBCAs was summarized by investigating the adjacent relationships of the surgical lesions with the external auditory canal and facial nerve.

**Results:**

Of the 20 patients, 16 were classified as Work type I and 4 as Work type II. The lesions were adjacent to the inferior wall cartilage of the auditory meatus in all children. Work type I lesions were located in the upper lateral aspect and were not adjacent to the facial nerve. Work type II lesions were located in the inferior-medial region of the facial nerve. The lesions were completely resected in all children. One patient experienced recurrence 3 months postoperatively because of a residual endochondral fistula. No patients developed facial paralysis or other complications.

**Conclusions:**

The inferior wall cartilage of the auditory meatus may help to the identify the initial lesion of the CFBCAs and can be regarded as a guiding anatomical structure. These lesions can be completely resected. For resection of Work type II first branchial cleft lesions, the surgical incision can be narrower, and can be precisely positioned with the assistance of endoscope.

**WHAT IS ALREADY KNOWN ON THIS TOPIC**

- Congenital first branchial cleft anomalies (CFBCAs) are rare congenital malformations of the head and neck and are great threats to children’s health.
- The most effective therapy for CFBCAs is surgery, which emphasizes complete resection of the lesion and protection of the facial nerve.

**WHAT THIS STUDY ADDS**

- The lesions were inside the area enclosed by the external auditory canal, mastoid process, and parotid gland.
- The lesions were closely related to the inferior wall cartilage of the auditory meatus.

**HOW THIS STUDY MIGHT AFFECT RESEARCH, PRACTICE OR POLICY**

- The inferior wall cartilage of the ear canal can be used as an anatomical guide for locating the initial segment of the CFBCAs, facilitating complete resection of the lesion.

## Introduction

Congenital first branchial cleft anomalies (CFBCAs) are rare congenital malformations of the head and neck, which are mainly characterized by the failure of the first and second branchial arches to fuse during the embryonic period, accounting for 1–9% of all branchial cleft malformations.^1^ Children with CFBCAs usually manifest as local swelling, pain or recurrent infection in the lateral neck below external auditory canal (EAC), ear canal effusion, an intraear mass, or ear canal stenosis in children, with a few cases complicated with the tympanic membrane.^2^ Due to its low morbidity and multiple clinical manifestations, some patients have no specific symptoms and are easy to be misdiagnosed. Currently, ultrasound (US), computed tomography (CT), magnetic resonance imaging (MRI), and ear endoscopy are used for clinical diagnosis. Surgical resection is the only effective radical treatment once diagnosis is confirmed. However, the complex anatomical conditions surrounding the lesion, which are closely related to the facial nerve, parotid gland, and EAC, may lead to postoperative complications, such as facial paralysis, salivary leakage, and stenosis of EAC. In most patients, lesions are incised and drained due to repeated infections before diagnosis, resulting in difficult to distinguish boundaries between the lesion and surrounding tissues, and recurrence after surgery, which remains a challenge for otolaryngologists. In addition, most patients are children, and psychological fear and traumatic stress increase due to repeated dressing changes and operations. Therefore, this study aimed to investigate the clinical characteristics of 20 children diagnosed with CFBCAs who underwent surgical treatment in our hospital, to identify the adjacent relationship between the lesion and EAC, parotid gland and facial nerve was identified, and to provide new insight forprecision medicine.

## Methods

### Patients and data collection

This retrospective study included patients who underwent surgery for CFBCAs in our department between December 2018 and June 2022. The inclusion criteria were as follows: children with clinical diagnosis and histopathologically confirmed CFBCAs who were under 18 years of age at time of surgery. The exclusion criteria were as follows: other branchial cleft anomalies presenting with ear and/or neck lesions, such as the second branchial cleft anomalies. US, CT and MRI were used to make the diagnosis and identify the position of the auditory meatus inferior wall cartilage bedore surgery. Patients’ demographic characteristics and surgical information were collected from medical records.

### Surgical method

**Figure 1:**
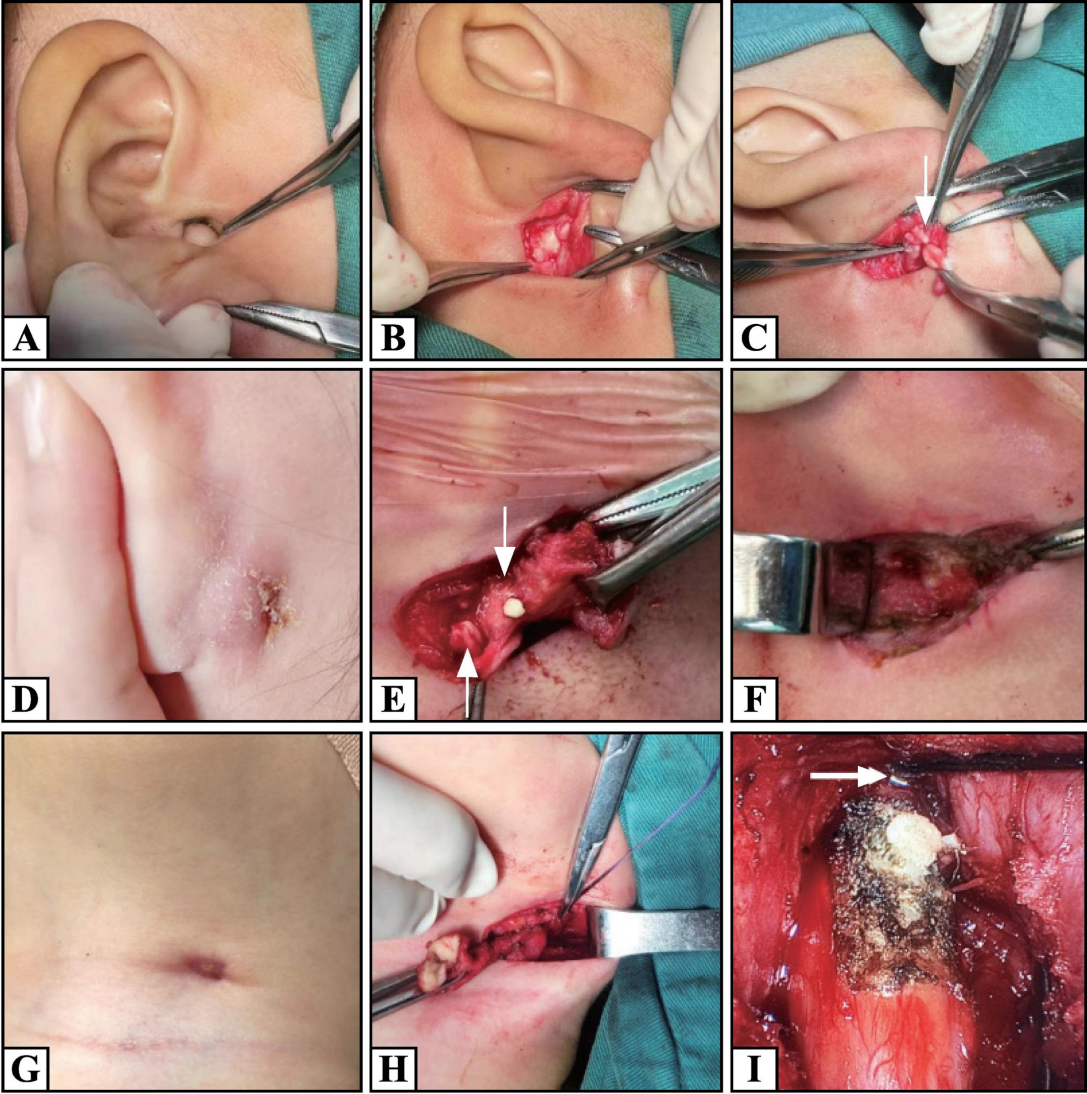
(A-C) For a Work type I cyst, a fusiform incision was made directly under direct visualization, exposing the inferior wall cartilage of the ear canal (arrow). (D-F) For Work type I fistula, a fusiform incision around the external fistula opening is used to incise the cartilage of the inferior wall of the ear canal (up arrow), and the bottom of the fistula is visible (down arrow). (G-I) For a Work type II cyst, a fusiform incision was made along the external orificium fistula, and a 0° endoscope was introduced horizontally into the mandibular angle. With the assistance of the endoscope, the end of the fistula was found at the cartilage of the lower wall of the ear canal (arrow).

All the patients underwent radical surgery during the static phase of inflammation (infection was controlled for at least 1 month). For Work type I cysts without infection, the posterior and inferior walls of the ear canal were located, and then a 2–2.5 cm arc incision was made along the posterior auricular sulcus toward the earlobe. The cartilage of the inferior wall of the ear canal was identified by careful separation of the subcutaneous tissue. The Cysts were completely removed by cutting the cartilage (figure 1A,B,C). The skin wound was carefully sutured to prevent connection between the surgical cavity and the ear canal. For the Work type I fistulas, a fusiform incision was made around the external fistula and scar tissue. The lesion and surrounding scar tissue were separated and bound posteriorly to the surface of the mastoid process, above the inferior wall cartilage of the ear canal, and anterior to the posterior edge of the parotid gland. The inferior cartilage wall of the ear canal was separated and incised to expose the fistula root. Complete resection of the lesion and part of the cartilage in this area was sufficient (figure 1D,E,F). Work type II fistulas are generally located at the lower edge of the parotid gland or the mandibular angle (figure 1G). First, a 1–1.5 cm fusiform incision was made at the neck of the fistula scar, and the scar tissue was separated to the horizontal position of the mandible (figure 1H). Second, a 0° endoscope (Karl-Storz Corporation, Tuttlingen, Germany) was used to assist in tracing the fistula tissue, and the root ended at the cartilage of the inferior wall of EAC (figure 1I). Simultaneously, a retroauricular incision was made to remove the initial part of the fistula in the cartilage of the inferior wall of the ear canal, and the operation method was the same as that for Work type I. A drainage tube was placed in the operative cavity for 48 hours. If the lesion involved the skin of EAC and caused injury, the ear canal skin was carefully sutured, and the ear canal was filled with a gelatin sponge for 2 weeks. All patients received antibiotic therapy for 1 week postoperatively.

All procedures performed in studies involving human participants were in accordance with the ethical standards of the institutional and/or national research committee and with the 1964 Helsinki Declaration and its later amendments or comparable ethical standards.

## Results

### General information

Totally, 20 children with CFBCAs were included, with 16 Work type I and 4 Work type II. The lesions were on the left side in 13 cases and right side in 7 cases; there were no cases with bilateral lesions (table 1). The mean age was 4.12±2.15 years old. Seven patients had cysts and the other 13 had fistulas. In 10 cases, incision and drainage were performed at the initial infection stage, of which 6 cases had at least one incision and drainage and 2 cases had seven incisions and drainages. The average incisions and drainages in every child was 1.5 times. Among all cases, 10 cases were diagnosed correctly for the first time, 3 were misdiagnosed as congenital preauricular fistula, 3 as furuncle of EAC, 2 as cases were diagnosed as sebaceous gland cysts, and 2 cases were diagnosed as parotid gland cysts. The primary symptoms were painless tumors around the ear with or without repeated redness, swelling, and pus discharge (12 cases); tumors in the ear canal (4 cases); and redness, swelling, and abscess in the neck (4 cases), all of which were located in the area above the bottom wall of EAC, below the upper edge of the hyoid, anterior to the submental, and posterior to the front edge of the sternocleidomastoid muscle .

**Table 1:** Relationship between Work classification and clinical characteristics of CFBCAs

Clinical features	Work type I (n=16)	Work type II (n=4)
Gender		
Male	9	2
Female	7	2
Lateral		
Left	10	3
Right	6	1
History of infection		
Yes	14	3
No	2	1
Course of the disease		
<1 year	3	2
≥1 year	13	2
Recurrence		
Yes	1	0
No	15	4
Postoperative facial paralysis		
Yes	0	0
No	16	4
The auricle was red and swollen after operation		
Yes	2	0
No	14	4

CFBCAs, congenital first branchial cleft anomalies.

**Figure 2:**
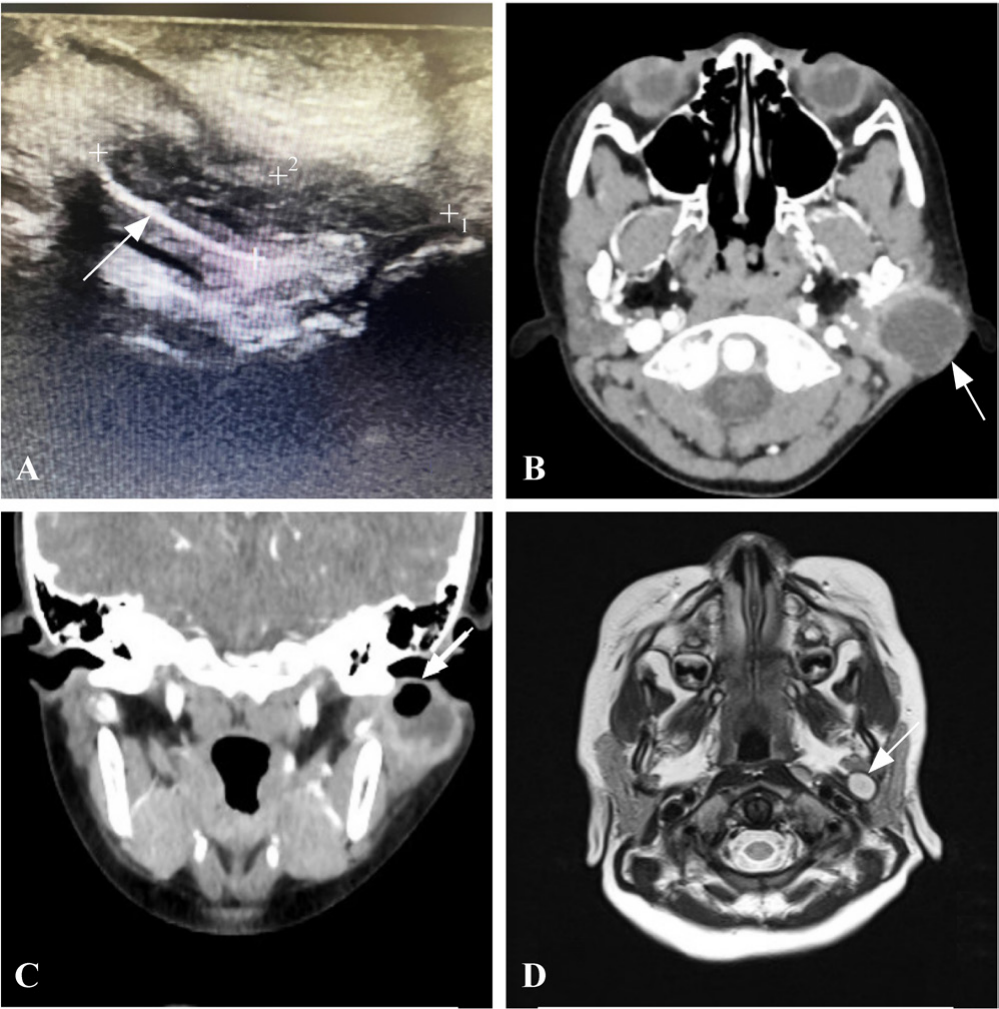
(A) The left external auditory canal can be seen in the inward downward hypoechoic, and the bottom of the lesion is the cartilage of the lower wall of the EAC (arrow); (B,C) he gas/liquid dark area can be seen posterolateral to the left parotid region, with an obvious annular enhancement at the margin, and the lower wall of the EAC at the bottom of the lesion (indicated by the arrow in C). (D) A cyst-like long T2 signal (arrow) was displayed in the medial aspect of the left parotid gland, lateral to the carotid sheath, with the outer edge close to the left parotid gland. EAC, the external auditory canal.

**Figure 3:**
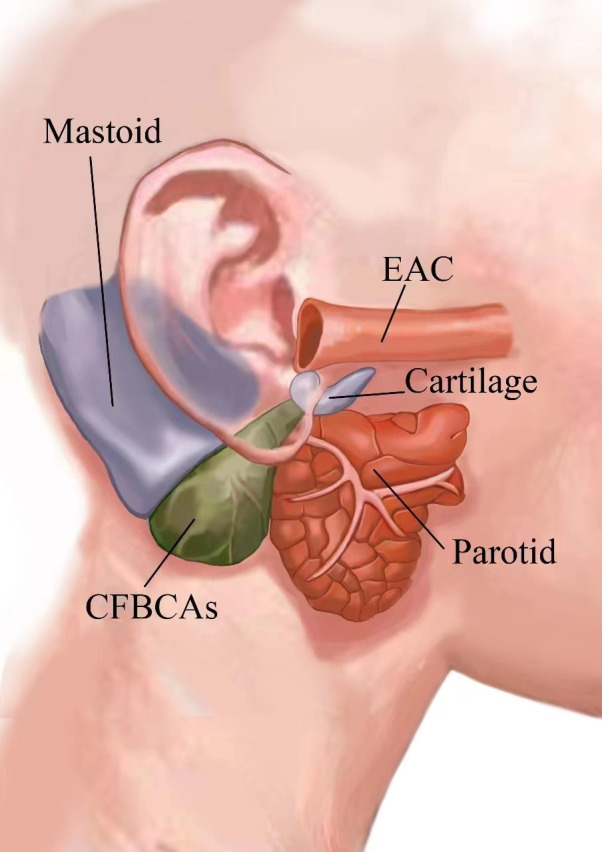
The anatomical relationship of CFBCAs to surrounding tissues. CFBCAs was below the external auditory canal, in front of the mastoid, behind the parotid, and the bottom of CFBCAs was located in the auditory meatus inferior wall cartilage. CFBCAs, congenital first branchial cleft anomalies; EAC, the external auditory canal.

Some typical imaging features in certain cases were as follows: (1) US: hypoechoic signal in the parotid gland and periauricular area, and the inferior wall cartilage of EAC at the bottom of the fistula (lesion), which was close to the lesion (figure 2A); (2) enhanced CT: low-density signals in the periauricular and parotid(figure 2B,C); (3) MRI: long T2 signal in the parotid/retroparotid sac(figure 2D).

### Intraoperative findings and follow up

We found that the initial segment of the CFBCAs was located at the inferior wall cartilage of the EAC and passed through the gap constructed by the mastoid, parotid glands, and the EAC (figure 3). In Work type I cases, the facial nerve was located in the anterolateral inferior part of the lesion, which is not adjacent to the lesion. Thus, we believed that there was no need to expose the facial nerves during the surgery. However, in a conventional retroauricular incision, the location of the posterior auricular artery needs to be confirmed to safely expose the cartilage and prevent injury during surgery. If the inflammatory scar adhesion is severe, tight sutures and ligation should be performed if injury occurs to avoid postoperative bleeding.

Redness, swelling, exudation, and pressure blisters were found 1 day after the operation due to obstruction of the drainage tubes in two children with Work type I lesions. When the drainage tube was placed again and the dressing changed for 2 weeks, no auricle deformity was found. The other patients were followed up for 6 months–2 years after surgery with no complications, such as parotid leakage, facial paralysis, or external auditory canal stenosis.

### Confirmed findings during reoperation after recurrence

**Figure 4:**
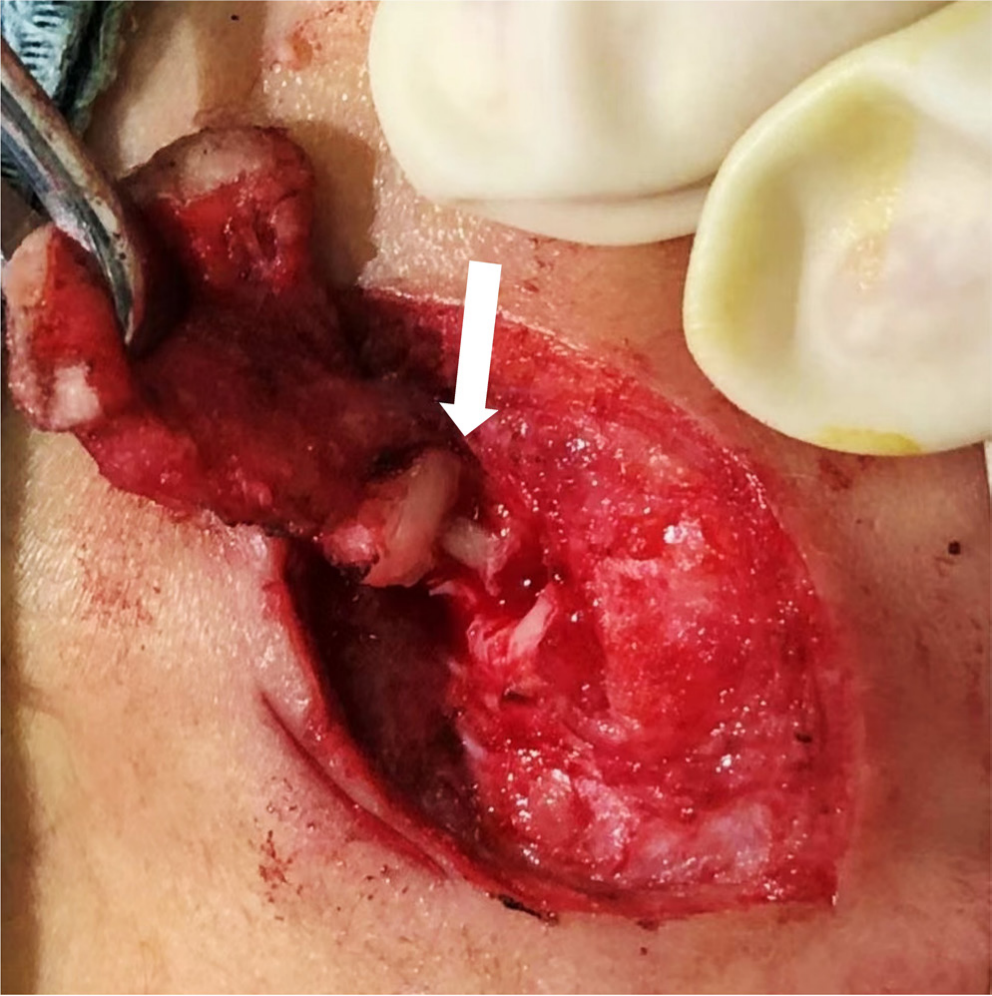
The cartilage of the inferior wall of EAC was dissected in the second operation of the recurrent patient, and residual fistula tissue is seen (arrow). EAC: the external auditory canal.

In the early stage due to inadequate experience, the inferior wall cartilage of EAC was exposed without cutting the cartilage to find the root of the fistula in one case with Work I type during surgery This case relapsed 3 months after surgery. During reoperation, the inferior wall cartilage of EAC was cut, and the residual fistula tissue was exposed (figure 4). No recurrence was observed during follow-up after complete resection.

## Discussion

CFBCAs are relatively rare, with an incidence of less than 8% among all branchial cleft malformations, and are caused by an incomplete closure of the first and second branchial arches during the embryonic period.^3^ The majority of CFBCAs occur in the area around the ear, especially in the retroauricular sulcus or the parotid area. Typical clinical manifestations include swelling and abscesses in the retroauricular and external fistula of the parotid gland.^4^ All patients in this study had lesions in the area around the ear. Because CFBCAs have no specific clinical manifestations and have relatively low morbidity, they are not easily diagnosed until there is recurrent redness, swelling, and pus, which can easily lead to a missed diagnosis or misdiagnosis.^5^ Many children with CFBCAs undergo repeated purulent resection and dressing changes before the final diagnosis, which can cause physical health problems.

Diagnosis of CFBCAs is highly dependent on the imaging modality. MRI and US are the most commonly used imaging methods.^6^ MRI findings of cyst-type CFBCAs demonstrated that the lesions were masses located around EAC, in the parotid gland, or at the posterior margin of the parotid gland, which were closely related to the inferior wall cartilage of EAC. MRI findings of fistula-type CFBCAs showed that the main focus was located posterior and inferior to EAC and extended laterally and inferiorly along the longitudinal axis of EAC. Work type I lesions mostly appeared outside the parotid gland, whereas Work type II lesions extended into the parotid gland, showing a tubular low-density signal. In some patients, the US showed that the bottom of the fistula was located in the cartilage of the inferior wall of EAC.

Currently, the most effective therapy for CFBCAs is surgery, which emphasizes complete resection of the lesion and protection of the facial nerve. There were two surgical incisions: a fusiform incision, including the external fistula and scar, and a fusiform incision around the lesion plus a straight incision extending to EACl.^7^ Regarding the location of lesions during surgery, management of EAC and facial nerve is particularly important to achieve complete resection of lesions to avoid recurrence.^8^ Triglia *et al* first proposed partial parotidectomy plus facial nerve dissection for patients with CFBCAs.^9^ It has been suggested that CFBCAs are often complicated by abnormalities in EAC, and the lesions can be identified within the cartilage of the posterior wall of the external auditory canal or the concha cavity. During surgery, part of the cartilage can be routinely removed to expose the basal part of the lesion.^10^ We found that, regardless of the Work type, the initial segment of the lesion was close to the inferior wall of EAC cartilage. Particularly for patients with repeated resection or recurrence resulting in local tissue scar adhesion and unclear structures, it is of great significance to find and identify this cartilage during surgery for complete resection of the diseased tissue. In this study, one patient with Work type I relapsed because the inferior wall cartilage of EAC was not exposed or incised during the first operation. The inferior wall cartilage of EAC was incised in the second operation to locate the residual fistula tissue, and complete resection was performed without recurrence during follow-up.

Postoperative facial paralysis is problematic for both patients and otolaryngologists. It has been reported that the incidence of permanent facial paralysis after CFBCAs ranges from 8% to 22%.^11 12^ For patients with a Work type I cyst, some researchers believe that the lesion is superficial to the facial nerve and there is no need to dissect it during surgery.^6 13^ However, it is also argued that Work type I cysts are mostly located above the main trunk of the facial nerve and have no relationship with it. However, Work type II lesions are mostly located in the parotid parenchyma, and fistulas are mostly located below the mandibular angle. The canal can pass through the lateral or medial side of the main trunk or between the main branches with a close connection to the facial nerve.^14^ D’Souza *et al* showed that 56.6% of lesions were located on the superficial surface of the facial nerve, 30.1% were located on the deep surface, and only 13.3% were located between the branches.^15^ In this study, it was found that the upper boundary of Work type I lesions was the inferior wall cartilage of the ear canal, the anterior boundary was the posterior margin of the parotid gland, and the posterior boundary was the mastoid region. The facial nerve was located in the anterior, lateral, and inferior tissues of the lesion, and the lesion was completely removed without dissecting the facial nerve, which is consistent with the aforementioned studies. For patients with Work type II lesions, the traditional method mostly uses the ‘Y’-shaped incision along the fistula or scar and extends to the outer ear canal, or the large ‘S’-shaped incision in the traditional parotid gland surgery. Such an incision can fully expose the facial nerve, parotid gland, and bottom of the diseased tissue; however, the wound surface is large, and the stretching or swelling of the facial nerve caused by excessive anatomical exposure also increases the risk of postoperative facial paralysis.^16^ Large wounds can cause more pain in children. In the present study, the fistulas were mainly located along the medial and inferior parts of the facial nerve. During surgery, a fusiform incision can be used to separate the fistula and scar tissue from the horizontal level of the mandible, and a 0° endoscope can be placed for tracking. Finally, the fistula ended at the inferior cartilage wall of EAC. According to the surgical requirements, a posterior auricular incision can be used to completely remove the fistula. Compared with traditional operative methods, our method involves a smaller incision, prevents unnecessary parotidectomy and excessive exposure of the facial nerve, and reduces the risk of postoperative facial paralysis, making it more appropriate for children. Surgery is suggested in children older than 1 year due to the immature development of the nervous system in children younger than 1 year.^17^ Overexposure or stretching of the facial nerve during surgery may cause complications such as facial paralysis. However, in our study, two patients had local redness and swelling of the auricle, exudation, and pressure-associated blisters on the local skin due to inadequate drainage, which were ameliorated after further drainage. Attention should be paid to the drainage of the surgical cavity, as well as to the resection of the lesion, thereby preventing edema of the auricle and ear canal skin caused by inadequate or no drainage, which also has a great impact on children. Ear canal stenosis and auricular deformities can occur in severe cases and should also be considered.

There are two limitations of this study. First, the number of cases, especially the number of Work type II lesion cases, was small; this is mainly due to the low incidence of the disease. Second, the follow-up time was short (6 months–2 years). Future studies should enroll more patients and extend the follow-up time for continuous improvement.

In conclusion, surgery is the only radical treatment option for CFBCAs. The inferior wall cartilage of the ear canal can be used as an anatomical guide for locating the initial segment of the CFBCAs, facilitating complete resection of the lesion. Endoscope-assisted resection of Work type II first branchial cleft lesions can narrow the surgical incision and reduce the risk of sialorrhea and facial paralysis.

10.1136/wjps-2023-000645.supp1 Supplementary data

## Data Availability

Data will be made available on request. The authors confirm that the data supporting the findings of this study are available within the article.
