# Supplementary material for: Significance of the auditory meatus inferior wall cartilage in the surgical treatment of congenital first branchial cleft anomalies in children
Source: World J Pediatr Surg. 2023 Nov 9;6(4):e000645. doi: 10.1136/wjps-2023-000645 (PMC10649883; doi:10.1136/wjps-2023-000645)
Supplement: Supplementary data [file wjps-2023-000645supp001.pdf]

Surgical procedure step by step:
Example 1:

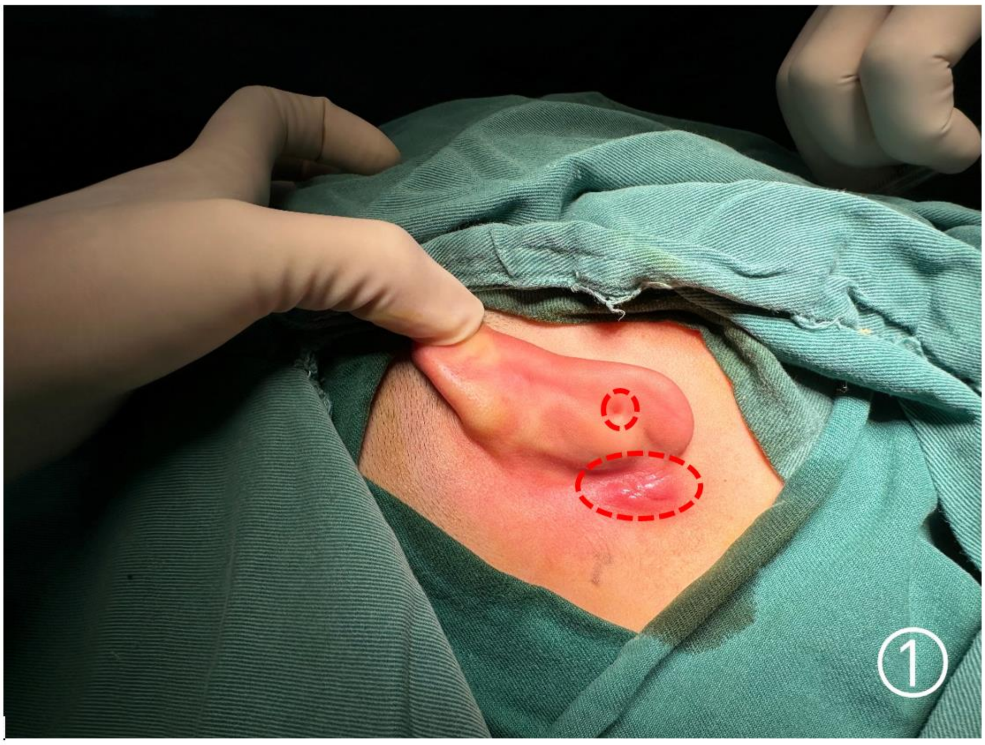

Figure 1: A fusiform incision is made at the location of the red lesion

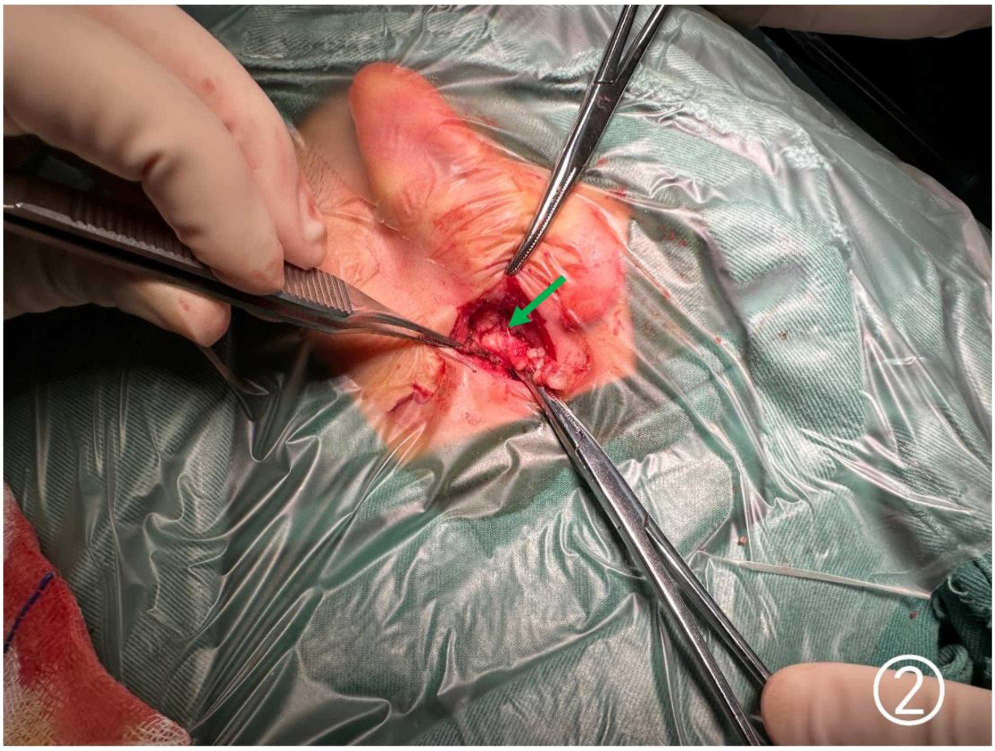

Figure 2: Cut the skin and subcutaneous tissue to expose the lesion（the green arrow represents CFBCAs）

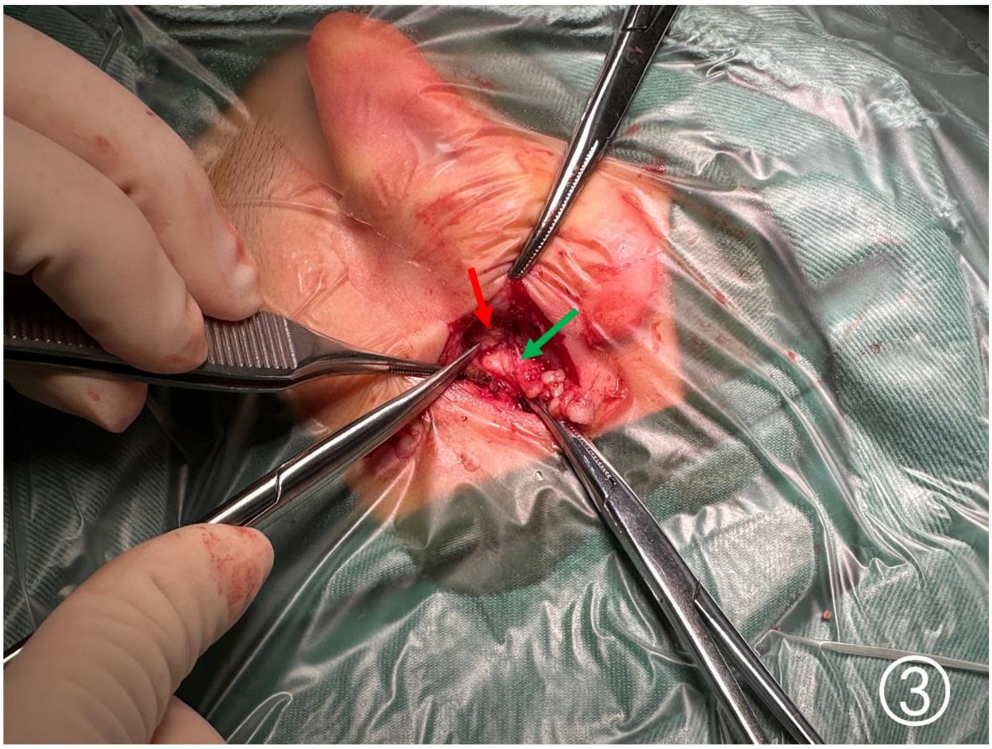

Figure 3: Continue to separate along the lesion（the green arrow represents CFBCAs，the red arrow represents the cartilage of the inferior wall of EAC）

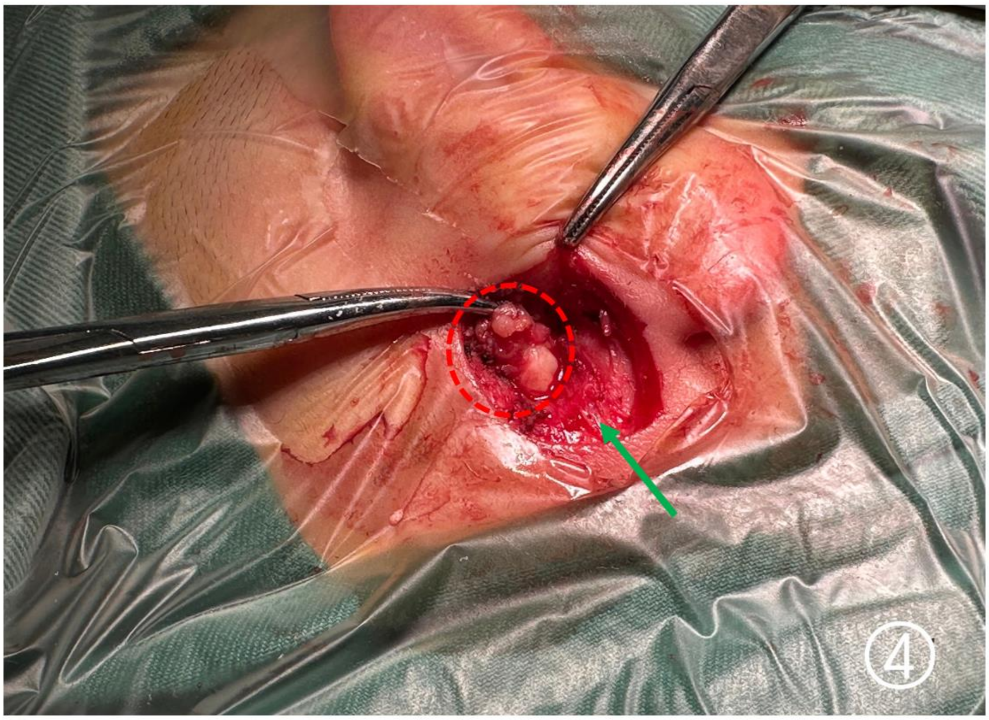

Figure 4: Continue to separate along the lesion（the red arrow represents CFBCAs，the green arrow represents posterior edge of the parotid gland）

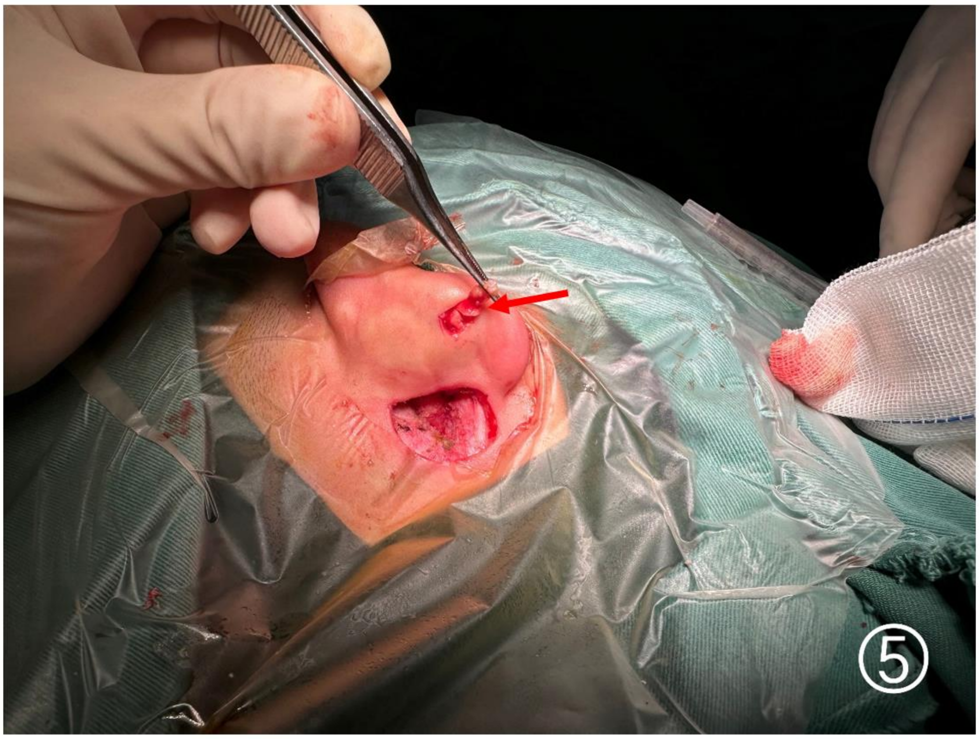

Figure 5: After complete removal of the lesion（the red arrow represents the small foramen on the pinna, which is not connected to the lesion）

Example 2:

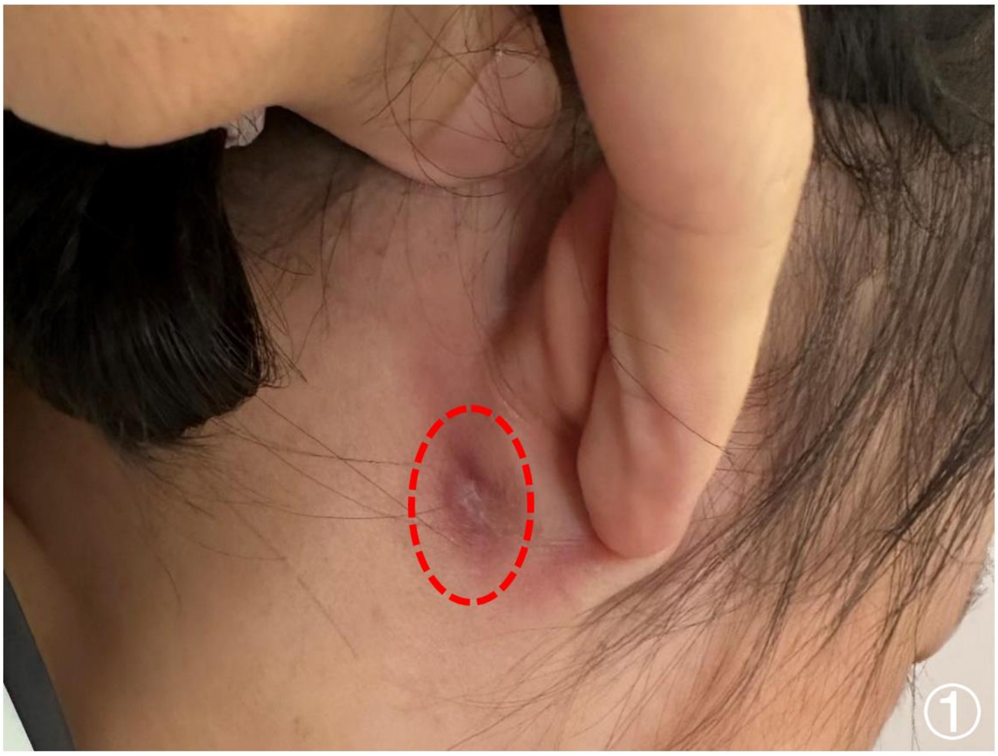

Figure 1: A fusiform incision is made at the location of the red lesion

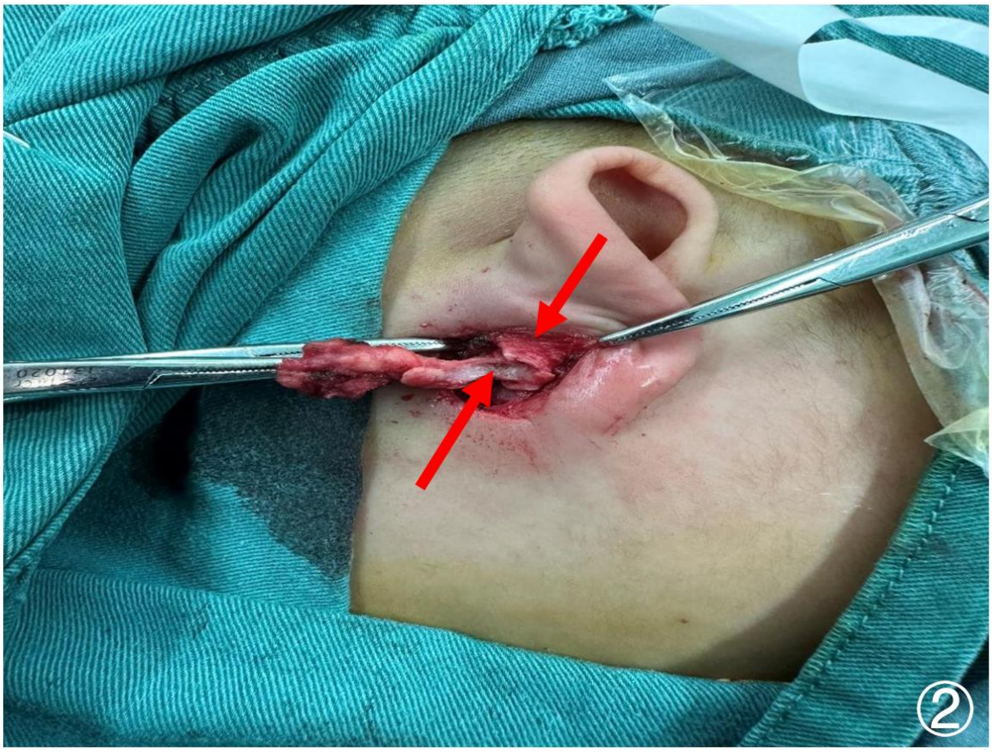

Figure 2: Cut the skin and subcutaneous tissue to expose the CFBCAs(up arrow), the

cartilage of the inferior wall of the ear canal(down arrow).

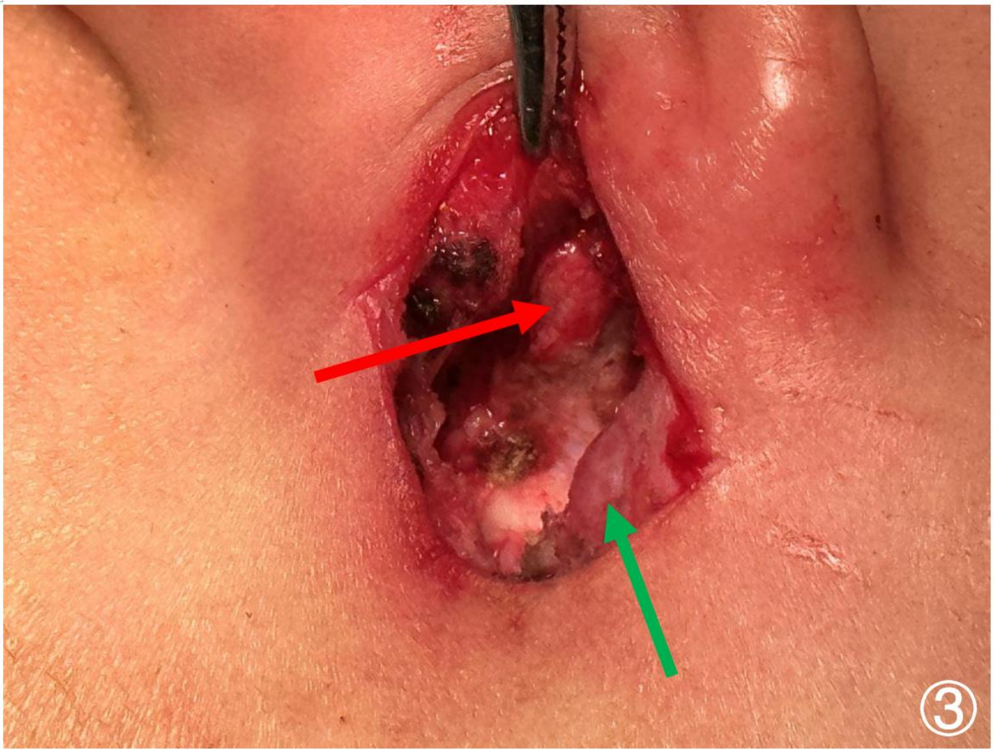

Figure 3:After complete removal of the lesion（the red arrow represents EAC skin exposed after removal of cartilage，the red arrow represents the posterior edge of the parotid gland）

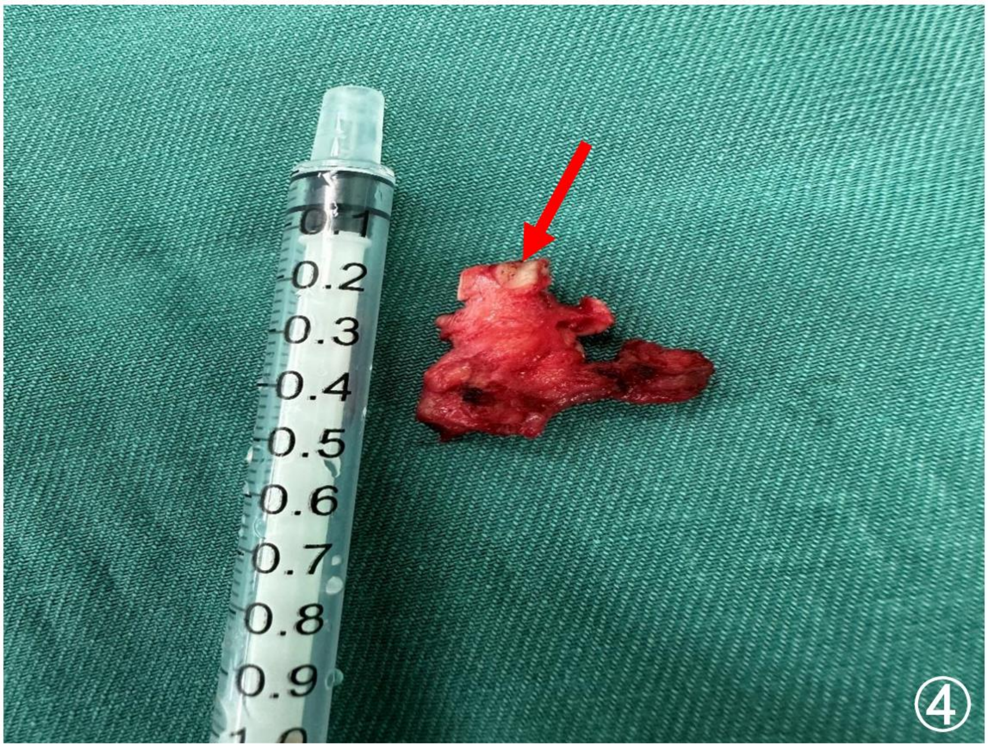

Figure 4，The lesion（the red arrow represents the cartilage of the inferior wall of EAC）。
